# Quality and reliability of sarcopenia-related videos on BiliBili and TikTok: a cross-sectional content analysis study

**DOI:** 10.1186/s12889-025-26154-x

**Published:** 2026-01-12

**Authors:** Hanchi Dong, Yirou Gong, Zihan Zhao, Keyan Wang, Xincheng Zhang, Fujian Ji

**Affiliations:** Department of Gastrointestinal and Colorectal Surgery, China-Japan Union Hospital of Jilin University, Changchun, China

**Keywords:** Public health, Quality and reliability, Sarcopenia, Social media

## Abstract

**Background:**

In recent years, the prevalence of sarcopenia has risen, frequently arising as a complication of other diseases and contributing to poor prognoses. Nonetheless, public awareness of this condition remains limited. Concurrently, video platforms such as BiliBili and TikTok, which reach billions of users worldwide, have become major channels for disseminating and accessing health information. This study systematically evaluates the quality and reliability of sarcopenia-related short videos on these two platforms and underscores the public health significance of this content.

**Methods:**

We conducted searches on October 3, 2025, using the keyword “sarcopenia” on the video-sharing platforms BiliBili and TikTok, ultimately collecting 256 relevant videos. After extracting basic information, we assessed the quality and reliability of each video using the Global Quality Score (GQS) for general quality and the modified DISCERN (mDISCERN) instrument for informational reliability.

**Results:**

Compared to BiliBili, TikTok showed significantly higher engagement metrics for likes, comments, shares, and views (all p* < 0.05, with rank-biserial r effect sizes ranging from − 0.40 to -0.53), whereas no significant difference was observed for favorites (p* = 0.168, rank-biserial *r* = -0.10). The median (interquartile range) of GQS and mDISCERN scores were both 3.00 (2.00–3.00) on BiliBili, while 3.00 (3.00–4.00) and 4.00 (3.00–4.00) on TikTok. Moreover, it was observed that videos from physicians in related fields and patient-shared videos both scored highly on TikTok; however, patient videos were very few (*n* = 3), so this difference is not generalizable. Spearman correlation analysis revealed no significant correlation between video variables and the scores of GQS and mDISCERN.

**Conclusion:**

Videos on the TikTok platform are of higher quality and reliability, and also exhibit stronger engagement. Moreover, videos uploaded by medical professionals generally have better quality than those from other sources, but they account for a low percentage. People should exercise caution when perusing videos about sarcopenia.

**Supplementary Information:**

The online version contains supplementary material available at 10.1186/s12889-025-26154-x.

## Introduction

Sarcopenia is a systemic skeletal muscle disorder defined by reduced muscle mass with low strength and/or impaired function [1]. It arises as part of aging or secondarily from chronic diseases, persistent inflammation, and treatment-related stressors. Established risk factors include inadequate protein and energy intake, endocrine and metabolic disturbances (e.g., insulin resistance and alterations of hormonal axes), chronic inflammation, and mitochondrial dysfunction [2–4]. Additional contributors encompass neuromuscular degeneration, organ dysfunction, physical inactivity, and drug toxicity, such as from corticosteroids and cytotoxic agents. Sarcopenia compromises physical function and quality of life and is associated with increased infection rates, postoperative complications, prolonged hospitalization, metabolic and immune dysregulation, and higher mortality [5–7]. Despite its clinical significance, substantial gaps in awareness persist—even among healthcare professionals, including physicians and nurses [8–10]. In some communities, sarcopenia’s status as a distinct disease entity is still debated, further impeding recognition [11]. These factors impose a considerable societal burden and underscore the need to enhance both public and professional awareness of sarcopenia.

Amid rapid economic and technological advances—particularly in digital communication—social media platforms have become a vital channel for public access to health information. The reliance on digital sources for health information is widespread; for instance, a 2018 survey indicated that nine out of ten American adults used the internet, and 75% of them sought medical or health-related information online [12]. In 2020, as the pandemic spread, many individuals were compelled to isolate. A cross-sectional study in China found that the internet was the most common source from which participants obtained COVID-19 knowledge [13]. Compared with traditional information channels, videos on social media have gained prominence because they are convenient, have low barriers to access, and offer strong visual appeal. Platforms such as BiliBili and TikTok attract substantial user engagement, making them major online sources of information. In 2022, BiliBili reported 92.8 million daily active users, 326 million monthly active users, and an average of 3.4 billion daily video views [14]. Meanwhile, TikTok is available in more than 150 countries and regions, has over 1 billion users, and has been downloaded more than 200 million times in the United States alone [15]. While online health information has improved public health literacy and fostered proactive health management, this digital paradigm also poses notable challenges. The unregulated nature of these platforms means information quality is highly variable, raising concerns about credibility and the potential for misinformation to influence health behaviors [16, 17]. Furthermore, the comprehensibility of information is crucial; major health institutions, such as the National Institutes of Health and the American Medical Association, recommend that patient education materials be written at a Grade 6 reading level to ensure broad accessibility [18]. Critically, the quality of information consumed directly relates to health outcomes. Patients who better understand the causes, pathophysiology, treatment, and prevention of their condition are more empowered to participate in and adhere to management plans, potentially leading to improved treatment outcomes [19]. Research shows that vaccine-related misinformation on social media can intensify vaccine hesitancy and potentially raise infection rates associated with emerging COVID-19 variants [20]. Such misinformation can also affect healthcare professionals’ decisions; for example, Warner et al. reported that cancer misinformation on social media substantially influences nurses’ health behaviors and care decisions [21]. Consequently, ensuring the accuracy and reliability of digital health information has become a critical public health priority. This has spurred the development of standardized tools, such as the Global Quality Score (GQS) and modified DISCERN (mDISCERN) instrument, to objectively assess the quality of online health information [22–24].

Previous studies have evaluated the quality of short videos across platforms for various disease categories, including respiratory and digestive conditions and orthognathic surgery [14, 25–27]. However, findings consistently show that video quality and reliability are frequently inadequate [14, 27]. Despite the volume of sarcopenia-related content on social media, no study to date has systematically evaluated its quality. This represents a significant knowledge gap, given the condition’s underdiagnosis and the public’s reliance on these platforms for health information. This persistent disconnect between video quality and platform popularity motivated us to examine sarcopenia-related videos. Despite sarcopenia’s prominence in health communication on social media, rigorous quality assessment is lacking. To address this gap, we conducted the first systematic evaluation of sarcopenia-related video quality on mainstream Chinese short-video platforms (BiliBili and TikTok) using validated instruments—the GQS and mDISCERN. This study aimed to: (1) quantify and compare the quality and reliability of sarcopenia-related videos on BiliBili and TikTok; and (2) identify video characteristics associated with higher quality scores. The findings will inform strategies for enhancing the quality of health information on social media and guide the public toward more reliable sources on sarcopenia.

## Methods

### Search strategy, data extraction, and classification

This cross-sectional study collected the top 150 videos from BiliBili and TikTok on October 3, 2025, sorted by each platform’s “comprehensive” ranking algorithm using the keyword “sarcopenia” in simplified Chinese (“肌肉减少症”) (details undisclosed by the platform). The sample size of 150 videos per platform was determined based on a pilot observation and considerations of analytical feasibility. Preliminary searches and scrolling behavior simulated by the research team indicated that the relevance and completeness of video content pertaining to the search term significantly diminished beyond the first several pages of results (approximately corresponding to the top 100–150 videos), with increased repetition of content and a higher prevalence of only marginally related material. This threshold (*N* = 150) was therefore adopted to capture the core set of prominent and accessible content likely to be encountered by a typical user performing a search, while maintaining a manageable scope for in-depth quality assessment.

We selected the platform-default “comprehensive” ranking for two primary, study-specific reasons. First, this ranking reflects what an average user encounters when performing a search, as most do not alter default sort settings. Thus, sampling from this output captures the information landscape that is most immediately and widely accessible to the public, which aligns with our study’s aim to assess the quality of content that typical viewers would find [28, 29]. Second, the “comprehensive” ranking is a composite algorithm that factors in relevance, engagement, recency, and author authority, thereby providing a broad, platform-curated snapshot of prominent content on the topic.

**Fig. 1:**
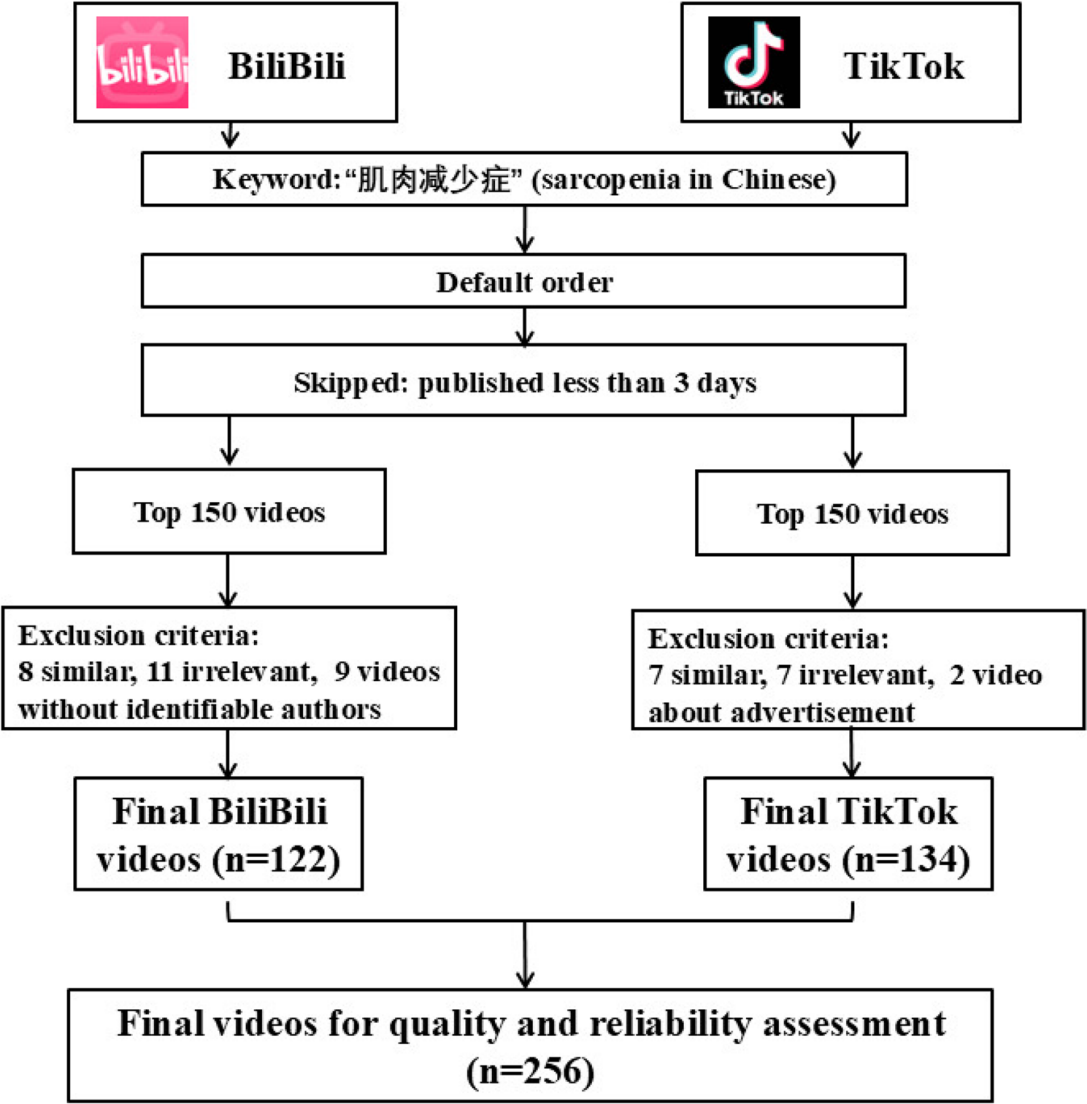
Search strategy and video filtering program

Prior to independent scoring, the two raters underwent a calibration exercise to ensure consistency. This involved jointly assessing 20 videos (not included in the final sample) using the GQS and mDISCERN instruments, discussing scoring decisions until a consensus on interpretation was reached. Searches were performed between 08:00 and 12:00 UTC to minimize diurnal fluctuations in content. To minimize bias from personalized recommendations, data collection was conducted using fresh accounts while logged out, with browser cache and cookies cleared, and the virtual location set to Changchun, China. We then screened the videos, with the detailed workflow shown in Fig. 1. Videos published less than 3 days prior to the collection date were excluded from analysis. This criterion was applied to ensure that the collected videos had sufficient time to accumulate stable, organic viewership and engagement metrics, which are essential for a fair assessment of their impact and audience reception. Newly uploaded videos might not yet have been fully disseminated by platform algorithms or reached a representative audience, potentially skewing the data [30]. To ensure the reproducibility of our search results, the analysis was based on the video content and metadata as they existed on the collection date. If a video later became private or was deleted, we retained all metadata that had been successfully captured for analysis. After applying the eligibility criteria, a total of 256 videos were included in the final analysis (BiliBili: *n* = 122; TikTok: *n* = 134). Restricting our analysis to the top 150 videos served two purposes. First, both platforms use search algorithms that prioritize topic relevance, ensuring that most sarcopenia-related videos appear at the top of the results. This is important because, when the number of results is large, some videos may be irrelevant or only marginally related to the topic. Second, viewers typically focus on the highest-ranked results rather than scrolling through the entire list [31, 32]. Subsequently, we extracted core video characteristics, including source, content, duration (seconds), engagement metrics (likes, comments, shares, favorites, and views), and days since publication. We then categorized videos by content and by source for further analysis. Detailed information is provided in Table 1.

**Table 1:** Detailed content of classification

Video sources	
Doctors in directly related fields	Including physicians practicing in geriatrics, rehabilitation medicine, endocrinology, and the clinical nutrition department.
Doctors in other fields	Including other specialists.
Patients	Including patients with sarcopenia.
Individual science communicators	Including non-professional, independent creators who produce and disseminate science-related content on digital platforms without formal institutional affiliation or credentialing.
Social organizations	Including social organizations that focus on health.
News agencies	Including TV stations and the media center.
**Video content**	
Disease knowledge	Including manifestation, anatomical, pathological, epidemiological, and basic research related to sarcopenia.
Disease treatment	Including treatment measures for sarcopenia.
Disease prevention	Including preventive measures for sarcopenia.
Case reports and news	Including patient reports, patient clinic videos, and news about sarcopenia.

### Video assessments

The quality and reliability of the videos were assessed using the GQS for general quality and mDISCERN for informational reliability. Although originally designed for written health information, both GQS and mDISCERN have been widely adapted and validated in prior studies for assessing video-based health content, demonstrating strong content validity in this context [12, 33]. Their adaptation is justified because they evaluate core dimensions of information quality—such as clarity, accuracy, sourcing, and balance—that are media-agnostic. For this study, the instruments were applied with explicit consideration of the video medium. We focused the assessment on the informational content conveyed through audio narration, on-screen text, and visuals. To account for temporal and production aspects that could affect viewer comprehension, such as pacing, audio-visual synchrony, and clarity of presentation, these factors were integrated into the operational definitions of the scoring criteria. For instance, a point was only awarded for clear source citation if references were presented audibly, visually on-screen, or in the video description with sufficient duration and clarity for a viewer to note. Similarly, GQS scores considered whether the video’s structure and presentation aided or hindered understanding.

The quality of the videos was assessed using the GQS, a commonly used 5-point Likert scale [34]. The scale ranges from 1 (poor quality) to 5 (excellent quality), with intermediate points representing generally poor, moderate, and good quality, as detailed in Supplementary Table S1. mDISCERN, an enhanced version of the DISCERN tool, assesses the reliability and completeness of information. The DISCERN instrument is reliable and valid for evaluating the quality of written consumer health information, enabling patients and healthcare professionals to distinguish quality within a large body of mixed-quality information [35, 36]. Similarly, mDISCERN has demonstrated strong applicability and has been widely adopted in research [26, 37]. The mDISCERN scoring system comprises five questions. For each question, a “Yes” response earns 1 point and a “No” response earns 0 points; higher total scores indicate greater video reliability (see Supplementary Table S2 for details). For item 2 of the mDISCERN tool (‘Are valid sources cited?‘), we operationalized “valid sources” as the clear presentation of any of the following: on-screen text references, verbal citations, captioned URLs, or direct links in the video description to clinical practice guidelines or peer-reviewed literature. Two students with relevant professional backgrounds independently scored the videos. Inter-rater reliability was assessed prior to the resolution of discrepancies. For the ordinal scales (GQS and mDISCERN), we report weighted kappa (κ_w) with quadratic weights. For the nominal categorical variables (video source, content, and format), we report Cohen’s kappa (κ). The agreement was as follows: GQS κ_w = 0.82, mDISCERN κ_w = 0.85, video source κ = 0.92, video content κ = 0.89, video format κ = 0.95. Any discrepancies were adjudicated by an arbiter, and consensus was achieved.

### Statistical analysis

For the statistical analysis, we established a priori that within-platform comparisons (e.g., comparing different categories of videos within BiliBili, and separately within TikTok) would be the primary focus. Cross-platform comparisons (e.g., directly comparing BiliBili videos to TikTok videos) were considered secondary and exploratory, given the inherent differences in platform design and user base that complicate direct comparisons. For the categorical variable “video source”, the low-frequency categories (‘patients’, ‘news agencies’, and ‘social organizations’) were combined into an “Other” category for all inferential statistical tests to meet their assumptions. The complete distribution of the original source categories is available in Supplementary Table S3. Given the non-parametric distribution of our data, continuous variables are reported as median and interquartile range (IQR). Group comparisons were conducted using the Mann–Whitney U test for two independent groups and the Kruskal–Wallis test for comparisons across more than two groups. These non-parametric tests were selected because the Shapiro-Wilk test confirmed that all continuous outcome variables (GQS, mDISCERN, and engagement metrics) significantly deviated from a normal distribution (p* <0.05). For significant Kruskal-Wallis results, post-hoc Dunn’s test with Bonferroni correction was applied for pairwise comparisons. For Mann-Whitney U and Kruskal-Wallis tests, effect sizes were calculated as r = Z/√N and ε² = (H - k + 1)/(n - k), respectively, and are reported alongside 95% confidence intervals (CIs) for medians where appropriate. Inter-rater agreement was assessed using Cohen’s κ coefficients. Spearman’s rank correlation, selected due to the non-normality of the data, was used to examine correlations among video variables and between these variables and the assigned scores. To control the false discovery rate (FDR) across all correlation tests and post-hoc pairwise comparisons, the Benjamini-Hochberg procedure was applied with a critical value of *q* = 0.05. To evaluate the robustness of the results, we reclassified the sources on the TikTok platform and compared the engagement metrics and descriptive statistics of sarcopenia-related videos among different sources. Statistical significance was defined as *p* < 0.05. All statistical analyses were performed in IBM SPSS Statistics version 26.0 for Windows, and data visualization was carried out using GraphPad Prism version 8.0.1 for Windows.

## Results

### Video characteristics

Applying predefined inclusion and exclusion criteria, we selected 256 videos for data extraction and subsequent analysis. Table 2 presents the baseline characteristics of the study sample. Video duration differed significantly between the two platforms (p* < 0.001), with BiliBili videos being longer (median: 164.00 s) and TikTok videos shorter (median: 112.50 s), with a rank-biserial r of 0.28 (95% CI: 0.14, 0.42). Additionally, the number of days since publication differed significantly between the two platforms (p* = 0.015), with a rank-biserial r of 0.18 (95% CI: 0.05, 0.32). On TikTok, viewer engagement metrics—including likes, comments, shares, and views—were significantly higher (all p* < 0.05), with moderate to large effect sizes (rank-biserial r ranging from − 0.40 to −0.53), while favorites did not differ significantly (p* = 0.168). TikTok demonstrated superior engagement, evidenced by higher counts of likes, comments, favorites, shares, and views.

**Table 2:** Characteristics of the videos on bilibili and TikTok

Variables	Total (*n* = 256), median (IQR)	BiliBili (*n* = 122), median (IQR)	TikTok (*n* = 134), median (IQR)	Rank-biserial *r* (95%CI)	P*
Duration (seconds)	112.50 (66.00, 216.25)	164.00 (71.00, 346.25)	92.50 (65.00, 153.25)	**0.28 (0.14**,** 0.42)**	**< 0.001**
Days since published	392.00 (113.50, 872.25)	589.00 (119.00, 1069.25)	287.00 (112.50, 658.75)	**0.18 (0.05**,** 0.32)**	**0.015**
Likes	197.00 (35.75, 1940.25)	53.00 (20.00, 675.25)	448.50 (92.50, 4186.75)	**−0.40 (−0.52**,** −0.26)**	**< 0.001**
Comments	5.50 (0.00, 77.50)	0.00 (0.00, 17.50)	18.00 (3.25, 171.50)	**−0.53 (−0.64**,** −0.41)**	**< 0.001**
Favorites	114.50 (23.75, 919.50)	81.00 (23.25, 673.75)	147.00 (27.50, 1533.25)	**−0.10 (−0.23**,** 0.04)**	0.168
Shares	57.50 (6.00, 407.25)	13.00 (2.00, 128.25)	158.50 (26.50, 1807.50)	**−0.46 (−0.58**,** −0.33)**	**< 0.001**
Views	16742.00 (3513.50, 92378.25)	8604.50 (1446.75, 21993.50)	48823.00 (9247.50, 368872.00)	**−0.50 (−0.56**,** 0.06)**	**< 0.001**

* indicates significance after Benjamini–Hochberg correction for multiple comparisons (*q* < 0.05)

### Publisher characteristics

**Fig. 2:**
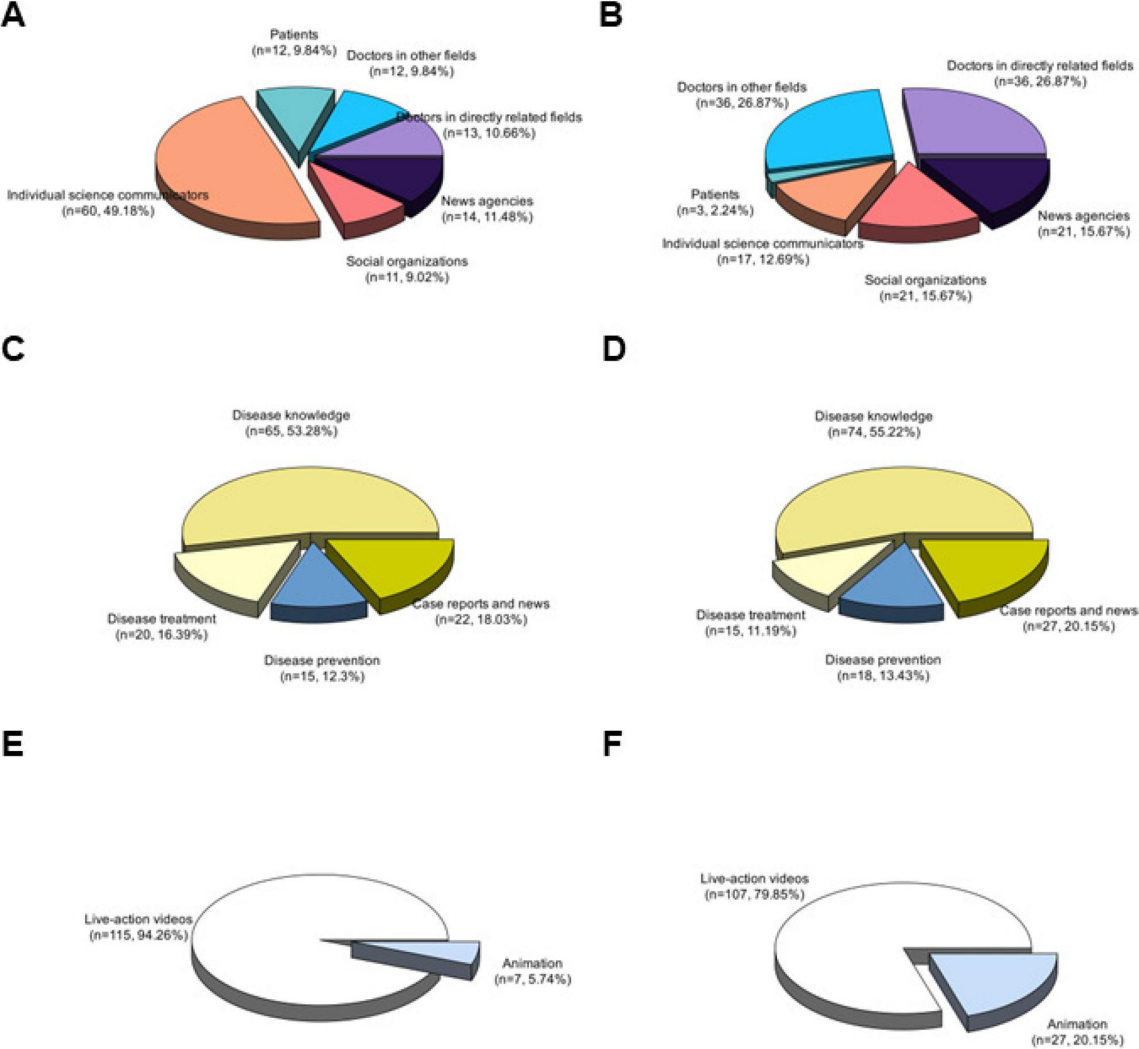
Percentage of videos according to video sources, video content, and video formats on BiliBili and TikTok. **A** Video sources on BiliBili. **B** Video sources on TikTok. **C** Video content on BiliBili. **D** Video content on TikTok. **E** also displays the analysis of video content) Video formats on BiliBili. **F** Video formats on TikTok

Among the video publishers included in the study, 97 (37.89%) were healthcare professionals, and 77 were individual science communicators. Notably, on BiliBili, individual science communicators constituted the largest group, surpassing healthcare professionals (Fig. 2). Social organizations and news agencies also held prominent positions, ranking among the top categories on both platforms. By contrast, patient-posted videos were scarce across both platforms. Surprisingly, on BiliBili, significantly higher engagement (likes, comments, favorites, and shares) and more views were observed for videos by individual science communicators than for those by doctors in directly related fields (Table 3). On BiliBili, significant differences were observed in likes, favorites, shares, and views across video sources (all p*< 0.05, ε² ranging 0.07–0.11). Similarly, on TikTok, content from individual science communicators received greater attention than that from other sources (Table 4). On TikTok, significant differences were observed in all engagement metrics (all p*< 0.01, ε² ranging 0.10–0.22; Table 4). In addition, we recombined the data from patients and individual science communicators on TikTok. After combining these sources, we compared engagement metrics and descriptive statistics of sarcopenia-related videos across different sources and found that the results remained unchanged (Supplementary Table S4).

**Table 3:** Engagement metrics and descriptive statistics of Sarcopenia-Related Videos, by Source, Content, and Format, on bilibili

Variables	Duration (seconds), median (IQR)		Days since published, median (IQR)		Likes, median (IQR)		Comments, median (IQR)	Favorites, median (IQR)		Shares, median (IQR)		Views, median (IQR)	
Video sources (*n* = 122)													
Doctors in directly related fields (*n* = 13)	163.00 (123.00,313.00)		668.00 (176.00,1328.00)		7.00 (5.00,41.00)		0.00 (0.00,0.00)	9.00 (2.00,40.00)		9.00 (1.00,25.00)		528.00 (158.00,1372.00)	
Doctors in other fields (*n* = 12)	238.00 (72.25,422.00)		260.50 (16.75,1048.75)		197.50 (33.00,932.75)		1.50 (0.00,13.75)	644.00 (67.25,2312.25)		119.00 (22.00,781.00)		4228.50 (2195.25,30198.75)	
Patients (*n* = 12)	112.00 (66.00,225.25)		686.00 (381.25,1215.75)		113.00 (73.50,294.25)		1.00 (0.00,2.50)	203.50 (54.00,369.25)		9.00 (5.25,29.75)		15563.00 (8653.25,22527.00)	
Individual science communicators (*n* = 60)	175.00 (69.00,410.00)		508.50 (86.25,1144.50)		153.50 (22.75,1272.50)		1.00 (0.00,59.75)	111.00 (29.25,1054.75)		26.50 (3.75,272.50)		9578.00 (1594.25,24807.75)	
Social organizations (*n* = 11)	144.00 (60.00,822.00)		812.00 (35.00,911.00)		43.00 (17.50,126.50)		0.00 (0.00,3.50)	48.00 (16.00,179.50)		2.00 (0.50,9.00)		15437.00 (4040.00,21238.00)	
News agencies (*n* = 14)	135.50 (54.00,185.25)		816.00 (354.75,820.00)		25.00 (21.50,40.50)		0.00 (0.00,0.00)	47.00 (24.00,119.75)		1.00 (0.25,10.50)		8604.50 (4654.75,14820.50)	
ε^2^ (95%CI)	**−0.01 (−0.04**,** 0.00)**		**−0.02 (−0.04**,** −0.01)**		**0.08 (−0.01**,** 0.16)**		**0.05 (−0.03**,** 0.10)**	**0.08 (−0.02**,** 0.16)**		**0.11 (−0.01**,** 0.20)**		**0.07 (−0.03**,** 0.15)**	
P*	**0.747**		**0.791**		**0.032**		**0.091**	**0.017**		**0.028**		**0.032**	
Video content (*n* = 122)													
Disease knowledge (*n* = 65)	173.00 (75.00,305.00)		592.00 (226.00,914.00)		33.00 (10.00,798.00)		0.00 (0.00,42.00)	48.00 (13.00,354.00)		11.00 (1.00,60.00)		6084.00 (561.00,21437.00)	
Disease treatment (*n* = 20)	95.50 (54.25,292.00)		905.50 (62.25,1156.75)		93.00 (39.00,266.00)		1.00 (0.00,2.50)	207.00 (76.25,734.25)		23.50 (2.00,120.75)		8553.00 (3514.50,27913.50)	
Disease prevention (*n* = 15)	214.00 (118.50,637.00)		156.00 (57.00,1146.00)		647.00 (51.50,1042.00)		2.00 (0.00,20.50)	840.00 (96.00,2841.50)		141.00 (36.50,517.00)		9889.00 (3611.50,28584.00)	
Case reports and news (*n* = 22)	127.50 (98.00,615.50)		555.50 (65.75,1263.25)		79.50 (28.00,410.00)		0.00 (0.00,17.25)	58.50 (18.75,229.00)		5.00 (2.00,40.00)		8604.50 (2473.50,15726.00)	
ε^2^ (95%CI)	**−0.01 (−0.03**,** 0.02)**		**−0.02 (−0.03**,** −0.02)**		**0.02 (−0.02**,** 0.10)**		**−0.02 (−0.02**,** −0.02)**	**0.08 (0.00**,** 0.17)**		**0.05 (−0.01**,** 0.14)**		**0.01 (−0.02**,** 0.07)**	
P*	**0.783**		**0.959**		**0.350**		**0.959**	**0.056**		**0.025**		**0.297**	
Video formats (*n* = 122)													
Live-action videos (*n* = 115)	165.00 (69.50, 336.50)		579.00 (96.00, 1021.00)		52.00 (20.00, 610.00)		0.00 (0.00, 10.50)	81.00 (23.50, 596.00)		12.00 (2.00, 115.00)		7839.00 (1520.50, 21705.50)	
Animation (*n* = 7)	156.00 (107.50, 382.50)		816.00 (504.50, 1383.50)		809.00 (18.50, 1813.50)		19.00 (0.00, 103.50)	877.00 (20.50, 2934.50)		270.00 (10.50, 1079.00)		16257.00 (5624.50, 26153.00)	
Rank-biserial r (95%CI)	**−0.04 (−0.28**,** 0.21)**		**−0.14 (−0.37**,** 0.12)**		**−0.21 (−0.47**,** 0.05)**		**−0.13 (−0.39**,** 0.16)**	**−0.22 (−0.46**,** 0.02)**		**−0.22 (−0.47**,** 0.04)**		**−0.22 (−0.46**,** 0.03)**	
P*	**0.934**		**0.165**		**0.387**		**0.245**	**0.370**		**0.199**		**0.520**	

*indicates significance after Benjamini–Hochberg correction for multiple comparisons (*q* < 0.05)

**Table 4:** Engagement metrics and descriptive statistics of Sarcopenia-Related Videos, by Source, Content, and Format, on TikTok

Variables	Duration (seconds), median (IQR)		Days since published, median (IQR)		Likes, median (IQR)		Comments, median (IQR)		Favorites, median (IQR)		Shares, median (IQR)		Views, median (IQR)	
Video sources (*n* = 134)														
Doctors in directly related fields (*n* = 36)	80.00 (56.50, 150.25)		237.50 (42.25, 731.25)		195.00 (70.25, 3289.75)		19.00 (5.00, 197.75)		46.00 (9.50, 1053.75)		63.50 (6.00, 1348.00)		24050.00 (5437.00, 297710.75)	
Doctors in other fields (*n* = 36)	75.50 (47.00, 126.75)		192.50 (84.50, 466.75)		1570.00 (261.50, 14144.00)		71.50 (12.75, 547.75)		312.50 (52.00, 3669.00)		339.00 (61.00, 6556.50)		212409.00 (22136.50, 1106852.00)	
Individual science communicators (*n* = 17)	88.00 (72.00, 186.00)		635.00 (271.00, 1152.00)		3518.00 (269.00, 24272.00)		102.00 (13.00, 926.00)		994.00 (99.00, 3476.00)		2016.00 (159.00, 10735.00)		282957.00 (36196.00, 1354785.00)	
Other^#^ (*n* = 45)	105.00 (69.00, 189.50)		291.00 (142.00, 721.50)		214.00 (49.50, 935.00)		5.00 (1.00, 18.00)		90.00 (21.00, 311.00)		87.00 (19.00, 324.50)		27343.00 (3902.50, 98905.00)	
ε^2^ (95%CI)	**0.06 (0.01**,** 0.18)**		**0.06 (0.01**,** 0.19)**		**0.12 (0.05**,** 0.26)**		**0.22 (0.11**,** 0.37)**		**0.10 (0.04**,** 0.21)**		**0.11 (0.05**,** 0.24)**		**0.11 (0.04**,** 0.24)**	
P*	**0.051**		**0.052**		**0.003**		**< 0.001**		**0.007**		**0.002**		**0.004**	
Video content (*n* = 134)														
Disease knowledge (*n* = 74)	92.50 (66.25,135.00)		271.00 (117.50,512.25)		408.00 (89.00,5698.25)		14.00 (2.25,151.50)		140.50 (37.50,2262.25)		198.50 (29.50,2384.00)		44258.00 (10736.00,430596.00)	
Disease treatment (*n* = 15)	117.00 (70.00,261.50)		291.00 (117.50,730.00)		1262.00 (144.50,7736.50)		37.00 (5.00,559.00)		311.00 (103.50,5087.50)		501.00 (111.50,6312.00)		108424.00 (11471.50,550365.00)	
Disease prevention (*n* = 18)	106.00 (77.75,153.25)		247.50 (45.50,1050.00)		738.00 (107.75.50,2801.25)		20.50 (4.75,88.25)		109.50 (20.25,679.75)		176.50 (74.50,1487.75)		40203.00 (5134.00,134963.00)	
Case reports and news (*n* = 27)	67.00 (53.00,185.50)		355.00 (104.00,712.50)		596.00 (93.50,2215.00)		21.00 (7.00,171.50)		154.00 (11.50,652.50)		62.00 (9.00,357.50)		70928.00 (7318.50,234195.00)	
ε^2^ (95%CI)	**0.01 (−0.02**,** 0.07)**		**−0.02 (−0.02**,** −0.02)**		**−0.01 (−0.02**,** 0.01)**		**−0.02 (−0.02**,** 0.00)**		**0.02 (−0.02**,** 0.07)**		**0.04 (−0.01**,** 0.11)**		**−0.01 (−0.02**,** 0.03)**	
P*	**0.446**		**0.979**		**0.889**		**0.924**		**0.446**		**0.378**		**0.889**	
Video formats (*n* = 134)														
Live-action videos (*n* = 107)	91.00 (62.50, 163.00)		269.00 (99.00, 641.00)		422.00 (89.00, 2814.50)		17.00 (3.00, 113.00)		129.00 (16.50, 1026.50)		133.00 (26.00, 1307.50)		44258.00 (8338.00, 251290.00)	
Animation (*n* = 27)	93.00 (67.00, 131.00)		357.00 (167.00, 721.50)		2633.00 (171.00, 13275.50)		89.00 (4.00, 537.50)		343.00 (70.50, 4075.00)		389.00 (60.50, 5123.00)		269042.00 (15100.00, 721318.00)	
Rank-biserial r (95%CI)	**−0.04 (−0.29**,** 0.19)**		**−0.14 (−0.39**,** 0.12)**		**−0.21 (−0.45**,** 0.04)**		**−0.13 (−0.41**,** 0.15)**		**−0.22 (−0.44**,** 0.03)**		**−0.22 (−0.48**,** 0.05)**		**−0.22 (−0.47**,** 0.06)**	
P*	**0.727**		**0.281**		**0.094**		**0.318**		**0.078**		**0.082**		**0.083**	

^#^ Since cells with fewer than 5 observations (*n* < 5) are presented for descriptive purposes only, no inferential statistical claims are made for these values, we have combined Patients

Social organizations and News agencies into “Other” category. * indicates significance after Benjamini–Hochberg correction for multiple comparisons (*q* < 0.05)

### Video content categorization

Figure 2 also displays the analysis of video content. Disease knowledge-related videos represented the largest share on both platforms—53.28% on BiliBili and 55.22% on TikTok. On BiliBili, treatment-related content was more prevalent than prevention-related content; on TikTok, the reverse held, with prevention-related videos exceeding treatment-related ones. Furthermore, on TikTok, case reports and news constituted the second-largest category after disease knowledge. Notably, despite constituting a small fraction of all content, disease-prevention videos on BiliBili garnered the highest engagement in likes, comments, favorites, shares, and views (Table 3). On BiliBili, disease prevention videos, though few, garnered the highest engagement in favorites and shares (p* = 0.056, ε² = 0.08 for favorites; p*= 0.025, ε² = 0.05 for shares; Table 3). By contrast, on TikTok, disease-treatment videos attracted greater attention though without significant differences across content categories (all p* > 0.05; Table 4). Regarding format, live-action videos dominated both platforms—94.26% on BiliBili and 79.85% on TikTok—as shown in Fig. 2. Nevertheless, a smaller subset of animation videos achieved relatively high engagement on both BiliBili and TikTok, measured by likes, comments, favorites, shares, and views though differences were not statistically significant (all p* > 0.05; Tables 3 and 4).

### Video quality and reliability assessment

**Fig. 3:**
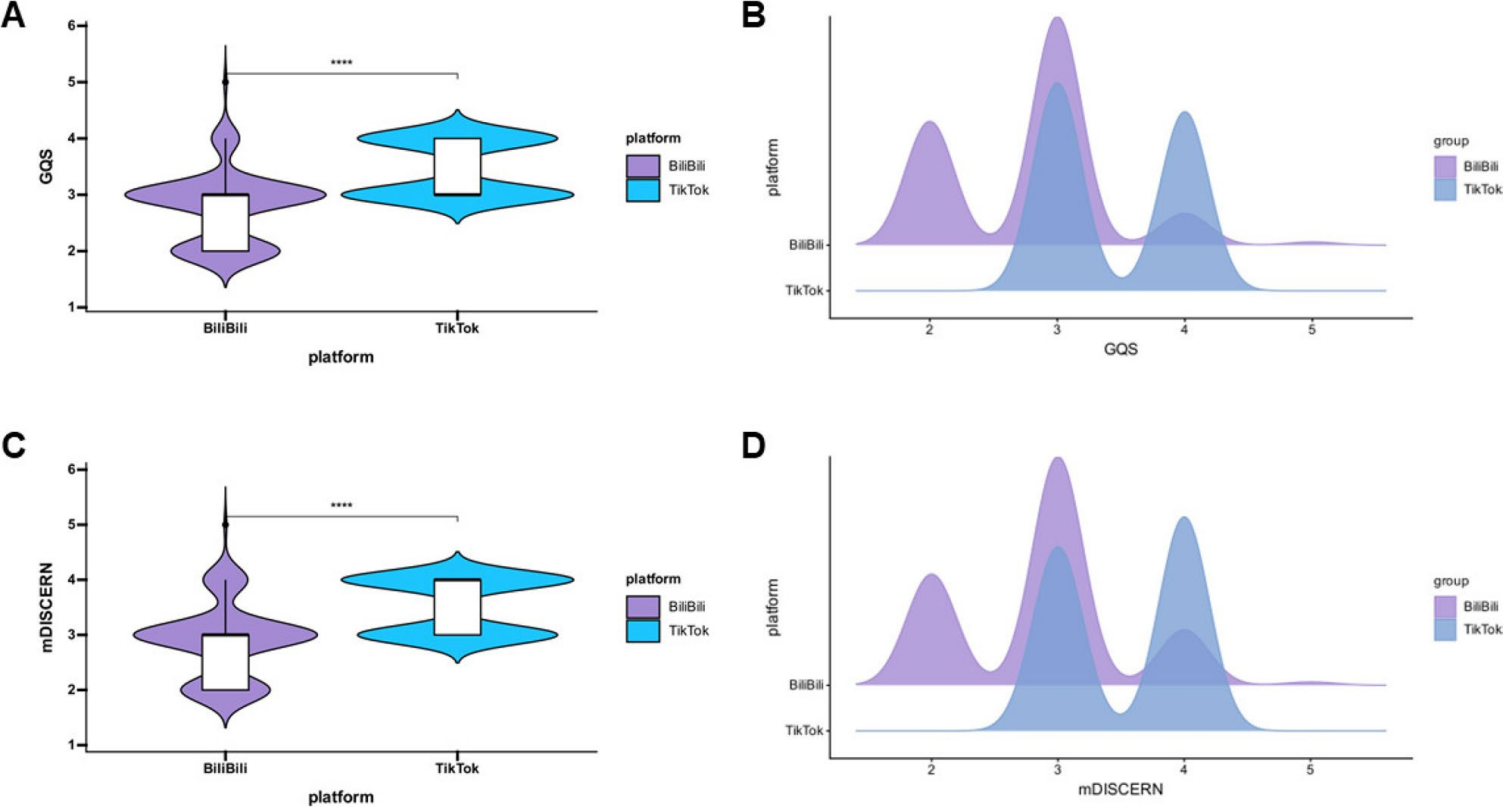
The GQS and DISCERN scores of videos related to sarcopenia on BiliBili and TikTok. **A** Comparison of GQS between BiliBili and TikTok videos. **B** Ridge plot showing the overall distribution of GQS. **C** Comparison of mDISCERN score between BiliBili and TikTok videos. **D** Ridge plot showing the overall distribution of mDISCERN score. “****” means p* < 0.0001. (Adjust the p-value without changing the shape of the image)

Video quality was assessed using the GQS, and reliability was evaluated with the mDISCERN instrument. BiliBili videos showed moderate quality and reliability, with median (IQR) scores of 3.00 (2.00–3.00) for both GQS and mDISCERN. By comparison, TikTok videos had a median (IQR) GQS of 3.00 (3.00–4.00) and a median (IQR) mDISCERN of 4.00 (3.00–4.00), indicating superior overall quality and reliability. Statistical analyses confirmed significant differences in both GQS and mDISCERN between platforms, with TikTok outperforming BiliBili (Fig. 3).

**Fig. 4:**
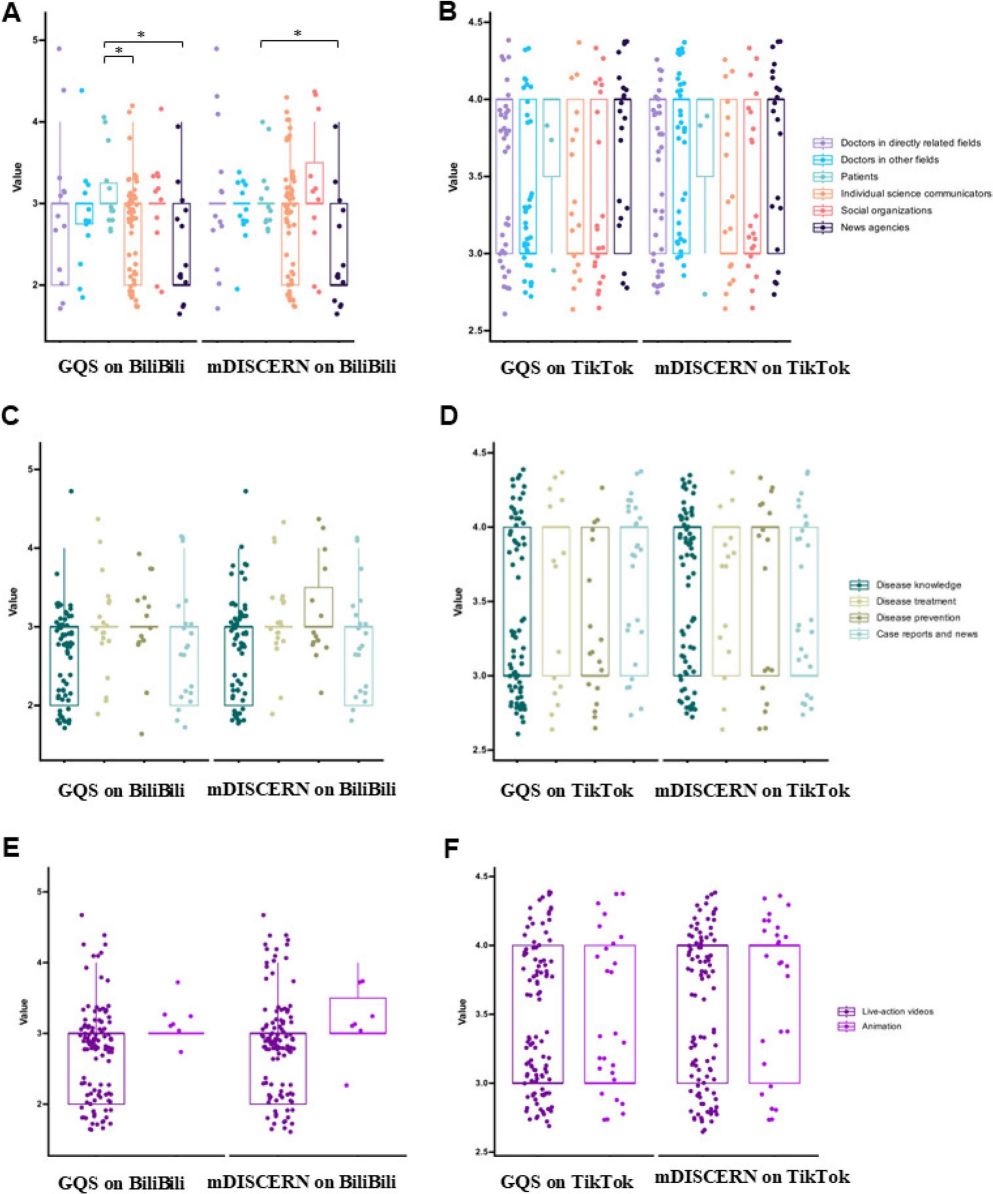
The GQS and mDISCERN scores of videos related to sarcopenia on BiliBili and TikTok. **A** The GQS and mDISCERN scores from different sources on BiliBili. **B** The GQS and mDISCERN scores from different sources on TikTok. **C** The GQS and mDISCERN scores from different content on BiliBili. **D** The GQS and mDISCERN scores from different content on TikTok. **E** The GQS and mDISCERN scores from different formats on BiliBili. **F** The GQS and mDISCERN scores from different formats on TikTok. “*” means p* < 0.05; “**” means p* < 0.01

Additionally, we conducted a statistical analysis of the GQS and mDISCERN across video sources, content, and formats, with detailed values reported in Table 5; Fig. 4. Assessing the influence of video source on content quality, we found that videos produced directly by physicians received higher ratings on both BiliBili and TikTok. On TikTok, content from physicians in directly related fields achieved GQS and mDISCERN scores of 4.00 (3.00–4.00), indicating strong endorsement of this material. By comparison, the corresponding scores on BiliBili were 3.00 (2.00–3.00) for GQS and 3.00 (3.00–3.00) for mDISCERN—good overall but slightly lower than on TikTok. No statistically significant differences were found across sources or content types on either platform after Benjamini–Hochberg correction (all p* > 0.05). In contrast, videos from physicians in unrelated fields, patients, individual science communicators, social organizations, and news agencies received comparatively lower ratings on both platforms, likely reflecting user expectations regarding the authority and expertise of information sources. Even so, ratings on TikTok were consistently higher. These findings suggest that strengthening the authority and professional rigor of video content—particularly in healthcare—is critical to improving user satisfaction and overall content quality.

**Table 5:** The GQS and mDISCERN score based on different video sources, content, and formats about sarcopenia on bilibili and TikTok

Variables		BiliBili			TikTok			
		GQS, Median (IQR)	mDISCERN score, Median (IQR)		GQS, Median (IQR)		mDISCERN score, Median (IQR)	
Overall score		3.00 (2.00, 3.00)	3.00 (2.00, 3.00)		3.00 (3.00, 4.00)		4.00 (3.00, 4.00)	
Video sources								
Doctors in directly related fields		3.00 (2.00,3.00)	3.00 (3.00,3.00)		4.00 (3.00,4.00)		4.00 (3.00,4.00)	
Doctors in other fields		3.00 (2.75,3.00)	3.00 (3.00,3.00)		3.00 (3.00,4.00)		4.00 (3.00,4.00)	
Patients		3.00 (3.00,3.25)	3.00 (3.00,3.00)		4.00 (3.50,4.00)		4.00 (3.50,4.00)	
Individual science communicators		3.00 (2.00,3.00)	3.00 (2.00,3.00)		3.00 (3.00,4.00)		3.00 (3.00,4.00)	
Social organizations		3.00 (3.00,3.00)	3.00 (3.00,3.50)		3.00 (3.00,4.00)		3.00 (3.00,4.00)	
News agencies		2.00 (2.00,3.00)	2.00 (2.00,3.00)		4.00 (3.00,4.00)		4.00 (3.00,4.00)	
ε^2^ (95%CI)		**0.07 (−0.02**,** 0.12)**	**0.06 (−0.03**,** 0.14)**		**0.04 (−0.03**,** 0.10)**		**−0.01 (−0.04**,** 0.01)**	
P*		**0.066**	**0.066**		**0.120**		**0.606**	
Video content								
Disease knowledge		3.00 (2.00,3.00)	3.00 (2.00,3.00)		3.00 (3.00,4.00)		4.00 (3.00,4.00)	
Disease treatment		3.00 (3.00,3.00)	3.00 (3.00,3.00)		4.00 (3.00,4.00)		4.00 (3.00,4.00)	
Disease prevention		3.00 (3.00,3.00)	3.00 (3.00,3.50)		3.00 (3.00,4.00)		4.00 (3.00,4.00)	
Case reports and news		3.00 (2.00,3.00)	3.00 (2.00,3.00)		4.00 (3.00,4.00)		3.00 (3.00,4.00)	
ε^2^ (95%CI)		**0.04 (−0.02**,** 0.12)**	**0.04 (−0.02**,** 0.12)**		**0.02 (−0.02**,** 0.08)**		**−0.02 (−0.02**,** −0.01)**	
P*		**0.108**	**0.108**		**0.223**		**0.897**	
Video formats								
Live-action videos		3.00 (2.00, 3.00)	3.00 (2.00, 3.00)		3.00 (3.00, 4.00)		4.00 (3.00, 4.00)	
Animation		3.00 (3.00, 3.00)	3.00 (3.00, 3.50)		3.00 (3.00, 4.00)		4.00 (3.00, 4.00)	
Rank-biserial r (95%CI)		**−0.35 (−0.61**,** −0.18)**	**−0.23 (−0.61**,** 0.23)**		**0.02 (−0.19**,** 0.24)**		**−0.12 (−0.32**,** 0.11)**	
P*		**0.079**	**0.259**		**0.832**		**0.283**	

* indicates significance after Benjamini–Hochberg correction for multiple comparisons (*q* < 0.05)

The type of video content significantly influences user satisfaction and content quality ratings. For disease knowledge and treatment, TikTok videos received high scores (4.00; IQR: 3.00–4.00), indicating strong user demand and satisfaction with practical, health-relevant content. By contrast, BiliBili scored lower (3.00; IQR: 2.00–3.00), suggesting room for improvement relative to TikTok. Disease prevention and case reports/news also emerged as prominent areas of user interest. For prevention-focused content, TikTok achieved a high mDISCERN score (4.00; IQR: 3.00–4.00), whereas BiliBili scored 3.00 (IQR: 3.00–3.50). Furthermore, case reports and news on TikTok attained a high GQS (4.00; IQR: 3.00–4.00). Collectively, these findings underscore the importance of prioritizing educational, practical, and directly health-related information in video production to address user needs and enhance overall content quality.

The video format materially influences user attention, content appeal, and perceptions of the GQS and mDISCERN. On TikTok, both live-action videos and animations achieved median GQS and mDISCERN scores of 4.00 (IQR: 3.00–4.00), suggesting that these formats effectively capture engagement, deliver high-quality viewing experiences, and are perceived as more reliable. In contrast, on BiliBili, live-action videos had a median score of 3.00 (IQR: 2.00–3.00) and animated videos 3.00 (IQR: 3.00–3.50). In terms of video formats, animation videos on BiliBili showed a trend toward higher GQS (rank-biserial *r* = −0.35, p* = 0.079) and mDISCERN (*r* = −0.23, p* = 0.259), though not statistically significant. On TikTok, format had no significant effect on quality or reliability scores (all p* > 0.05). While these values indicate reasonable performance, they reveal a notable shortfall relative to TikTok. This discrepancy may reflect platform-specific audience composition and consumption patterns, with TikTok users exhibiting a stronger preference for interactive and visually compelling formats. To optimize content appeal and user satisfaction, creators should employ more diverse and innovative video formats tailored to each platform’s audience preferences and viewing behaviors, thereby enhancing perceived content quality and reliability.

After adjusting for duration and days since publication, significant associations were observed on BiliBili between video sources, content, format, and the GQS (Table 6). Patient-sourced videos scored 0.49 points higher on the GQS than those from doctors in directly related fields (β = 0.49, 95% CI: 0.04, 0.93); content focusing on disease prevention scored 0.45 points higher than disease knowledge content (β = 0.45, 95% CI: 0.15, 0.75); and animated videos scored 0.50 points higher than live-action videos (β = 0.50, 95% CI: 0.08, 0.92). In contrast, no significant associations were found between video sources, content, or formats and GQS scores on TikTok. For the mDISCERN score, after controlling for duration and days since publication, only video content was significantly associated with mDISCERN scores on BiliBili (Table 7). Specifically, disease prevention content scored 0.46 points higher than disease knowledge content (β = 0.46, 95% CI: 0.11, 0.80). No significant associations were observed for video sources or formats with mDISCERN scores on BiliBili. Similarly, on TikTok, no significant relationships were found between video sources, content, or formats and mDISCERN scores.

**Table 6:** The association of video sources, content, and formats with GQS score on bilibili and TikTok

Factors	BiliBili		TikTok	
	β (95%CI)	*P*	β (95%CI)	*P*
Video sources				
Doctors in directly related fields	Reference		Reference	
Doctors in other fields	0.01 (−0.41, 0.43)	0.949	−0.16 (−0.41, 0.09)	0.215
Patients	0.49 (0.04, 0.93)	0.032	0.18 (−0.41, 0.78)	0.546
Individual science communicators	−0.18 (−0.51, 0.15)	0.280	−0.06 (−0.38, 0.25)	0.689
Social organizations	0.09 (−0.34, 0.53)	0.668	−0.09 (−0.37, 0.20)	0.556
News agencies	−0.20 (−0.61, 0.22)	0.347	0.24 (−0.06, 0.54)	0.119
Video content				
Disease knowledge	Reference		Reference	
Disease treatment	0.21 (−0.07, 0.48)	0.135	0.12 (−0.16, 0.41)	0.397
Disease prevention	0.45 (0.15, 0.75)	0.004	−0.04 (−0.30, 0.22)	0.759
Case reports and news	0.00 (−0.28, 0.28)	0.975	0.21 (−0.04, 0.46)	0.092
Video formats				
Live-action videos	Reference		Reference	
Animation	0.50 (0.08, 0.92)	0.021	−0.03 (−0.25, 0.19)	0.778

Note: adjusted for duration and days since published

**Table 7:** The association of video sources, content, and formats with mDISCERN score on bilibili and TikTok

Factors	BiliBili		TikTok	
	β (95%CI)	*P*	β (95%CI)	*P*
Video sources				
Doctors in directly related fields	Reference		Reference	
Doctors in other fields	−0.11 (−0.59, 0.37)	0.648	0.07 (−0.19, 0.33)	0.583
Patients	0.22 (−0.29, 0.73)	0.395	0.15 (−0.47, 0.76)	0.634
Individual science communicators	−0.21 (−0.59, 0.17)	0.278	−0.12 (−0.44, 0.21)	0.468
Social organizations	0.11 (−0.39, 0.61)	0.658	−0.13 (−0.43, 0.16)	0.369
News agencies	−0.46 (−0.93, 0.02)	0.058	0.05 (−0.26, 0.36)	0.756
Video content				
Disease knowledge	Reference		Reference	
Disease treatment	0.24 (−0.08, 0.55)	0.137	0.09 (−0.21, 0.39)	0.554
Disease prevention	0.46 (0.11, 0.80)	0.010	0.04 (−0.23, 0.31)	0.751
Case reports and news	0.01 (−0.31, 0.33)	0.956	−0.03 (−0.29, 0.23)	0.816
Video formats				
Live-action videos	Reference		Reference	
Animation	0.38 (−0.10, 0.86)	0.122	0.12 (−0.10, 0.35)	0.279

Note: adjusted for duration and days since published

### Spearman correlation analysis

**Fig. 5:**
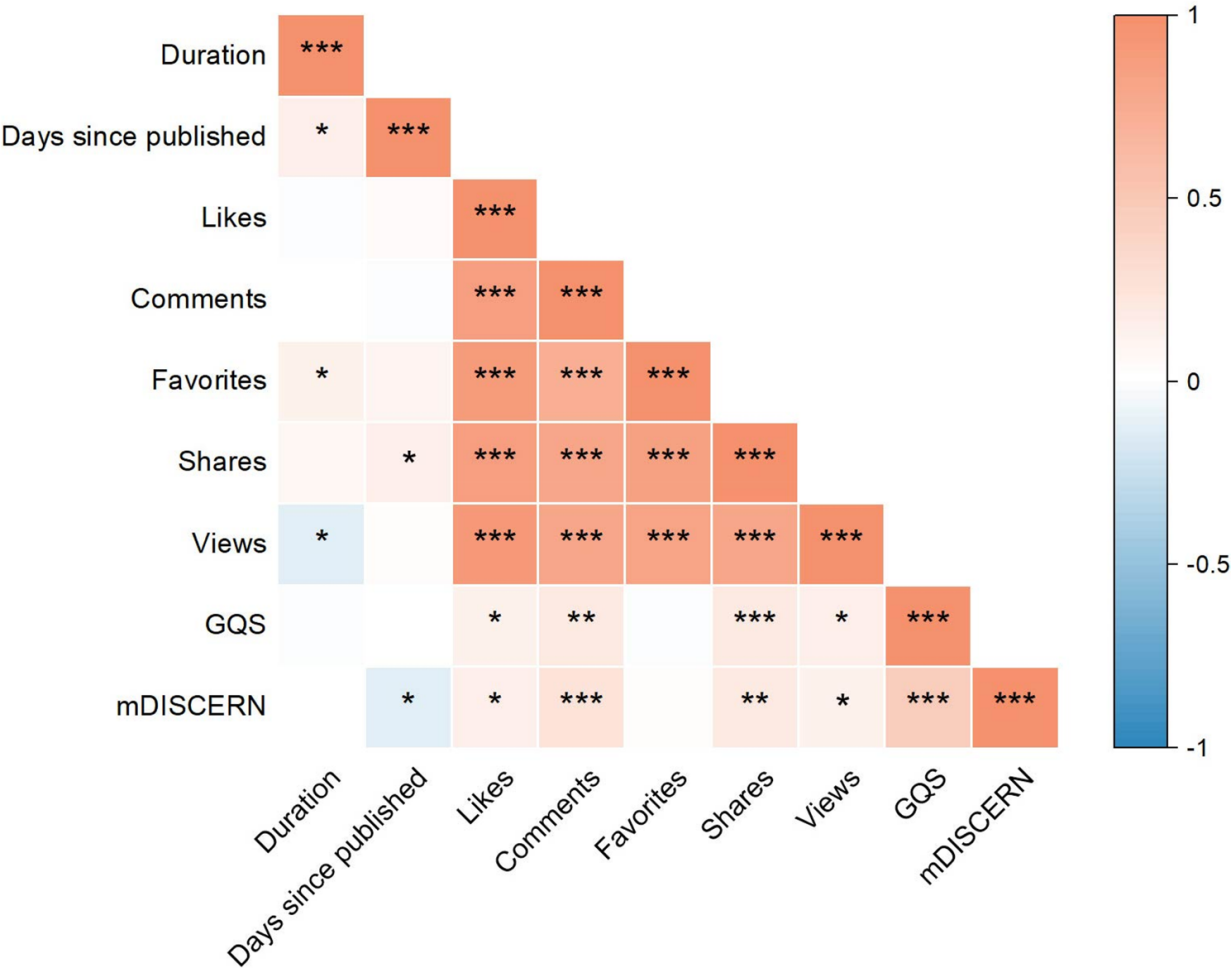
Spearman correlation analysis among different video variables, GQS and mDISCERN score concerning sarcopenia videos. “*” means p* < 0.05; “**” means p* < 0.01; “***” means p* < 0.001; “****” means p* < 0.0001

We employed Spearman correlation analysis to examine the relationships among various video variables, GQS, and mDISCERN scores in sarcopenia-related videos (Fig. 5). Significant positive correlations were observed between the number of comments and the numbers of likes, views, favorites, and shares, suggesting mutual reinforcement among user engagement behaviors. Strong positive intercorrelations were also evident among the numbers of likes, views, favorites, and shares, further supporting the interconnectedness of these engagement variables. Video duration showed weak correlations with engagement metrics (e.g., likes, views). Engagement metrics exhibited weak to negative correlations with the number of days since publication. No significant correlation was found between engagement metrics and GQS or mDISCERN scores.

## Discussion

In recent years, the incidence of sarcopenia has been rising, with increasing prevalence among younger individuals [38]. The prevalence of sarcopenia varies widely by age, setting, and diagnostic criteria; estimates in community-dwelling older adults typically range from ~ 5–25%, and are higher in clinical cohorts [39, 40]. Moreover, it frequently co-occurs with comorbid conditions, contributing to adverse outcomes and substantially reduced quality of life [41, 42]. Nevertheless, public awareness and understanding of this condition remain limited, and some even question its classification as a distinct disease. Given their broad reach and popularity, social media platforms (e.g., BiliBili and TikTok) are well-positioned to disseminate accurate health education. Accordingly, this study evaluates the necessity and effectiveness of disseminating health knowledge via social media, underscoring its practical relevance in addressing this public health challenge.

### BiliBili and TikTok as health information sources

Recently, social media has assumed an increasingly important role in healthcare, with approximately 80% of internet users seeking health information online [43, 44]. Among these platforms, video-based services such as BiliBili and TikTok have become particularly popular. These platforms host large and active user communities and frequent video updates, providing a solid foundation for our research. Prior work has shown that the quality and reliability of health-related videos require improvement, as demonstrated in studies on prostate cancer [45] and corroborated by similar findings in areas such as cataract and esophageal cancer [25, 46]. More critically, research indicates that video platforms can disseminate misleading health information. For example, a study on psoriasis by Simon M. Mueller et al. reported that nearly two-thirds of videos contained misleading or even hazardous content [47]. Patients who rely on inaccurate information from short videos may make erroneous health decisions and thereby expose themselves to risk. To promote the dissemination of accurate health knowledge, video platforms should strengthen their monitoring and review mechanisms. Such measures would help ensure high-quality, reliable content and protect users from potentially harmful or misleading information.

### Quality and reliability of the videos about sarcopenia

To our knowledge, the quality and reliability of online information about sarcopenia—particularly on video-sharing platforms—remain insufficiently characterized. Addressing this gap, our study demonstrates that while most videos achieve moderate quality and reliability, substantial improvement is warranted. Prior evidence indicates that content uploaded by professionals and professional organizations generally outperforms that produced by other sources in both quality and reliability [48]. In line with these observations, we found that videos created by medical professionals—especially doctors in directly related fields—provide greater instructional value, reflecting higher quality and reliability. This advantage likely stems from their stronger command of domain-specific knowledge, adherence to clinical guidelines, and familiarity with the latest research on sarcopenia. By contrast, nonmedical contributors, including patients and individual science communicators, often draw on personal experience and subjective interpretation, which can introduce bias. These findings underscore the central role of professional expertise in producing trustworthy medical video content, which aligns with studies on other diseases such as cervical cancer and Hashimoto’s thyroiditis [23, 49].

The observed differences in scores between platforms, while modest, invite consideration of how distinct platform environments might influence content presentation. This disparity may be attributed to fundamental differences in platform ecology and algorithmic curation. BiliBili, originating as a niche community for animation, comics, and games (ACG) culture, is known for a user base that highly values community interaction and creator personality. In contrast, TikTok, with its global reach and short-form video format, often emphasizes highly digestible content [50]. These differing contexts could shape content style, but our data does not establish a causal link to quality superiority. The most salient finding from our evaluation is that videos from doctors in directly related fields comprised only 19.14% (49/256) of our sample, a scarcity that likely depresses the overall quality of sarcopenia-related videos on both these platforms. Accordingly, the primary practical implication is that there is a pressing need to encourage physicians and medical institutions to produce higher-quality educational videos on sarcopenia, thereby improving public understanding and harnessing the potential of social media to advance population health. For consumers, this underscores the importance of critically evaluating source credentials when seeking health information on social media, regardless of platform.

The clinical and practical significance of the observed median score differences (typically 0–1 point on the 5-point GQS and mDISCERN scales) warrants explicit discussion. While statistically significant, the meaningfulness of such a difference must be contextualized within the instruments’ constructs. A one-point increase on the mDISCERN scale, for instance, could represent the inclusion of critical information previously omitted, such as stating the risks of a treatment or citing a clinical guideline. On the GQS, a one-point improvement might reflect a clearer structure or more balanced presentation that enhances viewer comprehension. Thus, even modest aggregate differences can signify a non-trivial shift in the informational integrity of content, potentially reducing the risk of viewer misunderstanding. However, translating this directly to a “clinical” impact on individual patient outcomes is complex. The effect is likely indirect and population-based: a systemic improvement in the average quality of publicly available information contributes to a healthier information ecosystem, which may support better public health literacy and more informed patient-clinician discussions over time [51–53]. Therefore, while the absolute score difference may seem small, it points to a meaningful gradient in information quality that has practical relevance for public health communication efforts aiming to elevate the baseline standard of social media health content.

### Correlation of video quality and reliability with video variables

Prior studies have indicated that video metrics such as likes, comments, and favorites may be associated with content quality and reliability [54, 55]. Our findings corroborate this observation. We observed strong positive correlations among likes, comments, favorites, shares, and views. However, the correlations between video quality and reliability and these engagement metrics were weak, consistent with the results reported by Zheng et al. [56]. This decoupling of engagement from quality underscores a critical challenge in health communication on social media: the metrics that drive visibility (likes, shares) are not necessarily aligned with those that ensure accuracy and depth [57]. Furthermore, our analysis revealed that video duration exhibited weak correlations with engagement variables, indicating that length may not be a primary driver of user interaction. Additionally, as the number of days since publication increased, engagement metrics (comments, likes, views) showed weak or even negative correlations, likely reflecting a natural decline in content popularity and algorithmic promotion over time, and underscoring the limited ‘lifespan’ of a video’s peak visibility. The lack of significant correlation between engagement metrics and GQS/mDISCERN scores further suggests a potential disconnect between content popularity and its informational depth or professionalism.

We also noted several noteworthy patterns. Despite their higher quality and reliability, videos uploaded by medical practitioners attracted less attention than those produced by individual science communicators. This disparity may arise because high-quality videos often employ formal language and specialized terminology, which can be less accessible to non-specialists. Furthermore, individual science communicators may excel at leveraging storytelling and emotional appeal—factors known to significantly boost user engagement but not always correlated with scientific accuracy [58, 59]. Recommendation algorithms may further reinforce these patterns: videos with higher levels of likes and interactions tend to be prioritized for recommendation over those with higher intrinsic quality. Moreover, animation appears to enhance engagement through a more vivid and appealing presentation. In our sample, a subset of animated videos—particularly on TikTok—garnered substantially more likes, comments, shares, and favorites. These results suggest that animation may be advantageous for disseminating medical and health information. Accordingly, we recommend that medical practitioners and institutions tailor content to audience preferences by using plain language and incorporating visual effects, animation, and other engaging formats. We also encourage platforms to strengthen their curation mechanisms and prioritize professional, high-quality content in search results to promote the dissemination of accurate information.

### Advantages and significance

With the rapid expansion of online social media—particularly the widespread adoption of video platforms—the quality assurance of health-related content has become a pressing concern. This study evaluates the quality and reliability of sarcopenia-related videos and is among the first investigations of its kind in China. Drawing on China’s two largest video platforms, BiliBili and TikTok, our multi-platform design mitigates single-platform bias and strengthens the representativeness and robustness of the findings. Methodologically, we employed two complementary rating instruments—the GQS and the mDISCERN—to assess video content across multiple dimensions, enabling a more comprehensive and nuanced appraisal. The results reveal considerable heterogeneity in content quality: many videos contain inaccuracies or lack adequate professional grounding, posing potential risks to public understanding and uptake of health information. These findings offer actionable guidance for improving the dissemination of accurate, high-quality health information. First, video platforms should enhance content oversight and implement rigorous quality assessment standards to ensure scientific accuracy. Second, platforms should refine search and recommendation algorithms to prioritize professional, high-quality health videos, thereby facilitating efficient access to trustworthy information. Third, experts and healthcare institutions should be encouraged to engage actively in content creation to produce materials that are both accessible to the public and aligned with professional standards, leveraging social media’s reach to improve health literacy. For example, the positive effects of β-Hydroxy-β-Methyl Butyrate (HMB) supplements on muscle mass and muscle strength index may be a high-quality content that the public enjoys and needs [60]. Concurrently, viewers—especially patients—should approach short-form health videos with caution and avoid making medical decisions based solely on such content; cultivating critical appraisal skills is essential. Ultimately, the dissemination of high-quality health information requires coordinated efforts among platforms, professional organizations, content creators, and audiences. Implementing the recommendations proposed herein can substantially improve the quality and accessibility of health information on short-video platforms and help build a stronger foundation for public health literacy.

### Limitations

While we are confident in our study, we acknowledge several limitations. Our analysis was restricted to Chinese social media platforms, which may introduce bias when generalizing findings to other linguistic contexts and underscores the need for cross-linguistic research. Additionally, focusing on BiliBili and TikTok, while justified by their massive user bases in China, means our findings may not generalize to other major health information channels. These include AI-powered platforms generating health content [61], global video platforms like YouTube which remain a primary source for procedural and condition-specific information [62], and dedicated medical websites often evaluated for reliability and readability [63]. The content dynamics, audience, and moderation on these platforms differ from the short-video, algorithm-driven ecosystems of BiliBili and TikTok. Second, the sample selection based on the top 150 videos from a single search, while justified by pilot observations of content relevance decay and feasibility, may not perfectly align with the precise scrolling limits of all health information seekers. This pragmatic threshold, though designed to capture prominent content, might have excluded some less-visible yet potentially relevant videos. Third, our use of the platforms’ default “comprehensive” ranking means our sample is inherently shaped by proprietary algorithms whose exact weighting of factors like engagement, recency, and authority is undisclosed. This “algorithmic bias” means we captured what the platforms deemed most visible, not necessarily a representative sample of all sarcopenia content. Furthermore, despite a rigorous calibration process, the use of GQS and mDISCERN instruments involves an element of subjective judgment. Although we achieved good inter-rater reliability, different assessors might score certain subjective aspects (e.g., “balance and bias” in mDISCERN) differently. In addition, as a cross-sectional study, our results reflect a single collection window; rankings and counts change over time. This design does not capture the “lifespan” of a video’s quality or popularity, nor does it account for how content might be updated or removed. Longitudinal tracking is needed to understand the dynamics of health information evolution on these platforms. What’s more, while this study assessed informational quality, it did not directly measure the real-world impact of the identified low-quality or misleading content on viewers’ health beliefs or behaviors. Such misinformation carries potential harms, including the delay of appropriate medical consultation or the adoption of ineffective or unsafe management strategies. Finally, although our sample size was adequate for the current analysis, a wider sampling would improve the generalizability of the findings. As platforms such as WeChat and Kwai increasingly incorporate video features, future work should broaden platform coverage to more comprehensively evaluate the quality of sarcopenia-related videos.

## Conclusions

This study assessed the quality of 256 sarcopenia-related videos on BiliBili and TikTok using the GQS and mDISCERN instruments. Across both platforms, median video quality/reliability was moderate; TikTok scores were slightly higher than BiliBili for GQS and mDISCERN. Favorites did not differ across platforms. Videos from medical professionals generally scored higher but represented a minority of the content; increasing the proportion of professionally produced videos may improve the overall information quality. Accordingly, medical professionals and institutions should recognize the growth of video-sharing platforms and produce high-quality sarcopenia content. In addition, these platforms should strengthen their monitoring and review systems, and patients should exercise caution when selecting videos on BiliBili and TikTok. Future research should develop and evaluate targeted interventions, such as training programs for healthcare content creators, and employ longitudinal designs to track the evolution of content quality and the impact of such initiatives over time.

## Supplementary Information

Supplementary Material 1

Supplementary Material 2

Supplementary Material 3

Supplementary Material 4

## Data Availability

The datasets used and analyzed during the current study are available fromthe corresponding author on reasonable request.
