# Supplementary material for: Quality and reliability of sarcopenia-related videos on BiliBili and TikTok: a cross-sectional content analysis study
Source: BMC Public Health. 2026 Jan 12;26:517. doi: 10.1186/s12889-025-26154-x (PMC12888518; doi:10.1186/s12889-025-26154-x)
Supplement: Supplementary file 1 — Supplementary Material 1 [file 12889_2025_26154_MOESM1_ESM.docx]

**Supplementary table 1. Global Quality Score (GQS) scoring standard. (Scoring ranges from 1 to 5)**

| **GQS description** | **Score** |
| --- | --- |
| Poor quality, poor flow of the site, most information missing, not at all useful for patients | 1 |
| Generally poor quality and poor flow, some information listed but many important topics missing, of very limited use to patients | 2 |
| Moderate quality, suboptimal flow, some important information is adequately discussed but others poorly discussed, somewhat useful for patients | 3 |
| Good quality and generally good flow, most of the relevant information is listed, but some topics not covered, useful for patients | 4 |
| Excellent quality and excellent flow, very useful for patients | 5 |
