# Supplementary material for: Quality and reliability of sarcopenia-related videos on BiliBili and TikTok: a cross-sectional content analysis study
Source: BMC Public Health. 2026 Jan 12;26:517. doi: 10.1186/s12889-025-26154-x (PMC12888518; doi:10.1186/s12889-025-26154-x)
Supplement: Supplementary file 2 — Supplementary Material 2 [file 12889_2025_26154_MOESM2_ESM.docx]

**Supplementary table 2. Modified DISCERN (mDISCERN) scoring standard. (1 point for answer ‘yes’, 0 point for answer ‘no’)**

| **Reliability Score** |  |  |  |
| --- | --- | --- | --- |
| 1. Is the video clear, concise, and understandable? | | | |
| 2. Are valid sources cited? | |  |  |
| 3. Is the content presented balanced and unbiased? | | | |
| 4. Are additional sources of content listed for patient reference? | | | |
| 5. Are areas of uncertainty mentioned? | | |  |
