# Supplementary material for: Quality and reliability of sarcopenia-related videos on BiliBili and TikTok: a cross-sectional content analysis study
Source: BMC Public Health. 2026 Jan 12;26:517. doi: 10.1186/s12889-025-26154-x (PMC12888518; doi:10.1186/s12889-025-26154-x)
Supplement: Supplementary file 3 — Supplementary Material 3 [file 12889_2025_26154_MOESM3_ESM.docx]

**Supplementary Table S3. Characteristics of the videos in accordance with sources, contents, and formats on TikTok.**

| Variables | Duration (seconds), median (IQR) | Days since published,  median (IQR) | Likes, median (IQR) | Comments, median (IQR) | Favorites, median (IQR) | Shares, median (IQR) | Views, median (IQR) |
| --- | --- | --- | --- | --- | --- | --- | --- |
| Video sources (n = 134) | | | | | | | |
| Doctors in directly related fields (n = 36) | 80.00 (59.50,142.75) | 237.50 (48.75,693.75) | 195.00 (78.75,2897.25) | 19.00 (5.00,179.25) | 46.00 (10.50,967.25) | 63.50 (6.00,1282.00) | 24050.00 (6043.00,262350.25) |
| Doctors in other fields  (n = 36) | 75.50 (47.00,120.25) | 192.50 (91.50,446.25) | 1570.00 (294.50,11912.00) | 71.50 (14.25,491.25) | 312.50 (54.00,3227.00) | 339.00 (69.00,3941.50) | 212409.00 (25051.50,1018266.00) |
| Patients (n = 3) | 135.00 (122.00,198.50) | 43.00 (35.00,587.50) | 20.00 (11.00,461.50) | 3.00 (2.00,108.00) | 11.00 (6.00,176.50) | 28.00 (14.00,80.50) | 1354.00 (724.50,36141.00) |
| Individual science communicators  (n = 17) | 91.00 (77.00,186.00) | 635.00 (271.00,1152.00) | 2730.00 (269.00,15026.00) | 86.00 (13.00,853.00) | 994.00 (99.00,2910.00) | 470.00 (159.00,6621.00) | 282957.00 (60796.00,1262838.00) |
| Social organizations  (n = 21) | 95.00 (66.00,119.00) | 271.00 (212.00,504.00) | 422.00 (199.00,1104.00) | 7.00 (1.00,18.00) | 162.00 (69.00,311.00) | 137.00 (39.00,501.00) | 44258.00 (27343.00,108424.00) |
| News agencies  (n = 21) | 136.00 (69.00,188.00) | 441.00 (127.00,784.00) | 90.00 (26.00,371.00) | 5.00 (1.00,12.00) | 44.00 (13.00,143.00) | 43.00 (13.00,228.00) | 10736.00 (3401.00,40203.00) |
| Video content (n = 134) | | | | | | | |
| Disease knowledge  (n = 74) | 92.50 (66.25,135.00) | 271.00 (117.50,512.25) | 408.00 (89.00,5698.25) | 14.00 (2.25,151.50) | 140.50 (37.50,2262.25) | 198.50 (29.50,2384.00) | 44258.00 (10736.00,430596.00) |
| Disease treatment  (n = 15) | 117.00 (70.00,261.50) | 291.00 (117.50,730.00) | 1262.00 (144.50,7736.50) | 37.00 (5.00,559.00) | 311.00 (103.50,5087.50) | 501.00 (111.50,6312.00) | 108424.00 (11471.50,550365.00) |
| Disease prevention  (n = 18) | 106.00 (77.75,153.25) | 247.50 (45.50,1050.00) | 738.00 (107.75.50,2801.25) | 20.50 (4.75,88.25) | 109.50 (20.25,679.75) | 176.50 (74.50,1487.75) | 40203.00 (5134.00,134963.00) |
| Case reports and news  (n = 27) | 67.00 (53.00,185.50) | 355.00 (104.00,712.50) | 596.00 (93.50,2215.00) | 21.00 (7.00,171.50) | 154.00 (11.50,652.50) | 62.00 (9.00,357.50) | 70928.00 (7318.50,234195.00) |
| Video formats (n = 134) | | | | | | | |
| Live videos (n = 107) | 91.00 (62.50, 163.00) | 269.00 (99.00, 641.00) | 422.00 (89.00, 2814.50) | 17.00 (3.00, 113.00) | 129.00 (16.50, 1026.50) | 133.00 (26.00, 1307.50) | 44258.00 (8338.00, 251290.00) |
| Animation (n = 27) | 93.00 (67.00, 131.00) | 357.00 (167.00, 721.50) | 2633.00 (171.00, 13275.50) | 89.00 (4.00, 537.50) | 343.00 (70.50, 4075.00) | 389.00 (60.50, 5123.00) | 269042.00 (15100.00, 721318.00) |

**Cells with n<5 are descriptive; no inferential claims.**
