# Supplementary material for: Quality and reliability of sarcopenia-related videos on BiliBili and TikTok: a cross-sectional content analysis study
Source: BMC Public Health. 2026 Jan 12;26:517. doi: 10.1186/s12889-025-26154-x (PMC12888518; doi:10.1186/s12889-025-26154-x)
Supplement: Supplementary file 4 — Supplementary Material 4 [file 12889_2025_26154_MOESM4_ESM.docx]

**Supplementary Table S4. Engagement Metrics and Descriptive Statistics of Sarcopenia-Related Videos, by reclassified Source on TikTok.**

| Variables | | Duration (seconds), median (IQR) | | Days since published,  median (IQR) | | Likes, median (IQR) | | Comments, median  (IQR) | | Favorites, median (IQR) | Shares, median (IQR) | | Views, median (IQR) | |
| --- | --- | --- | --- | --- | --- | --- | --- | --- | --- | --- | --- | --- | --- | --- |
| Video sources  (n = 134) |  | |  | |  | |  | |  | | |  | |  |
| Doctors in directly related fields(n=36) | | 80.00 (56.50, 150.25) | | 237.50 (42.25, 731.25) | | 195.00 (70.25, 3289.75) | | 19.00 (5.00, 197.75) | | 46.00 (9.50, 1053.75) | 63.50 (6.00, 1348.00) | | 24050.00 (5437.00, 297710.75) | |
| Doctors in other fields(n=36) | | 75.50 (47.00, 126.75) | | 192.50 (84.50, 466.75) | | 1570.00 (261.50, 14144.00) | | 71.50 (12.75, 547.75) | | 312.50 (52.00, 3669.00) | 339.00 (61.00, 6556.50) | | 212409.00 (22136.50, 1106852.00) | |
| Social organizations(n=21) | | 95.00 (62.00, 155.00) | | 271.00 (211.00, 568.00) | | 422.00 (185.50, 1104.50) | | 7.00 (0.50, 18.00) | | 162.00 (62.00, 325.50) | 137.00 (35.50, 531.50) | | 44258.00 (23565.00, 122177.50) | |
| News agencies(n=21) | | 136.00 (69.00, 189.50) | | 441.00 (124.50, 785.00) | | 90.00 (24.00, 375.00) | | 5.00 (1.00, 15.00) | | 44.00 (10.50, 174.00) | 43.00 (12.00, 308.50) | | 10736.00 (3281.00, 44790.50) | |
| Others^#^(n=20) | | 104.00 (77.00, 200.25) | | 521.50 (106.25, 1147.00) | | 1429.50 (145.00, 13384.50) | | 71.00 (7.75, 724.25) | | 513.00 (77.50, 2824.25) | 358.00 (119.75, 6067.75) | | 152944.50 (20508.50, 1216864.50) | |
| ε^2^ (95%CI) | | 0.04 (-0.01, 0.16) | | 0.02 (-0.02, 0.15) | | 0.09 (0.01, 0.23) | | 0.17 (0.08, 0.33) | | 0.06 (0.00, 0.21) | 0.06 (0.00, 0.21) | | 0.07 (0.01, 0.21) | |
| P* | | 0.075 | | 0.181 | | 0.018 | | **<0.001** | | 0.028 | 0.028 | | 0.026 | |

^#^ Since cells with fewer than 5 observations (n < 5) are presented for descriptive purposes only, no inferential statistical claims are made for these values, we have combined individual science communicators and patients into “Other” category. * indicates significance after Benjamini–Hochberg correction for multiple comparisons (*q* < 0.05).
